# An intrinsic synthesis parameter governing the crystallization of silico(zinco)aluminophosphate molecular sieves†

**DOI:** 10.1039/d1sc02431k

**Published:** 2021-06-30

**Authors:** Sung Hwan Park, Kingsley Christian Kemp, Jingeon Hong, Jung Gi Min, Suk Bong Hong

**Affiliations:** Center for Ordered Nanoporous Materials Synthesis, Division of Environmental Science and Engineering POSTECH Pohang 37673 Korea sbhong@postech.ac.kr

## Abstract

One of the most fundamental but yet unanswered questions in the synthesis of zeolites and zeolite-like materials is whether or not any parameter controlling the microporosity of the crystallized product from synthesis mixtures with feasible chemical compositions exists. Here we report that an experimentally optimized parameter (*ca.* 3.3 ≤ MOH/P_2_O_5_ ≤ 5.3, where M is alkali metal ions) is the criterion bringing about the successful formation of various high-charge-density silicoaluminophosphate (SAPO) and zincoaluminophosphate (ZnAPO) molecular sieves, without the aid of organic structure-directing agents. The materials obtained using this empirical concept include SAPO molecular sieves with CHA and LTA topologies, as well as a SAPO FAU/EMT intergrowth, and ZnAPO ones with CZP and SOD topologies. This study demonstrates the existence of an essential factor determining not only phase selectivity but also microporosity (0.3–2 nm) in the synthesis of zeotypes with charged frameworks which may offer interesting opportunities for more efficiently producing novel zeolite structures and/or compositions.

## Abstract 2

The existence of an essential parameter (*ca.* 3.3 ≤ MOH/P_2_O_5_ ≤ 5.3, where M is alkali metal ions used as structure-directing agents) determining phase selectivity and microporosity in the synthesis of zeotypes with charged frameworks is demonstrated.

## Graphical abstract

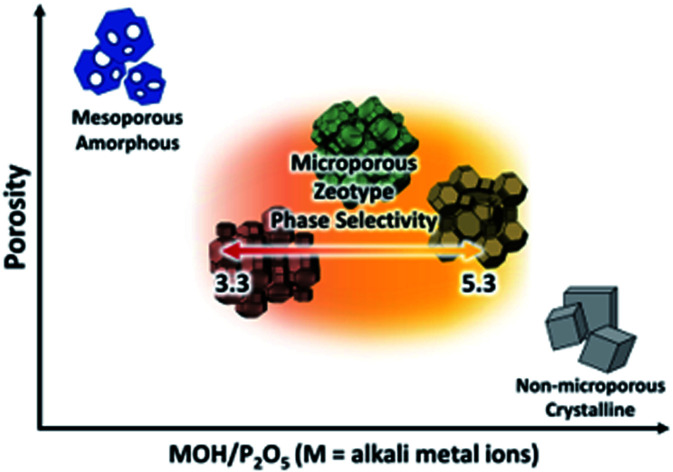

## 1 Introduction

Zeolites and molecular sieves are an important class of industrial catalysts and adsorbents.^1–4^ Although they are complex in structures and compositions, and crystallized under sophisticated synthesis conditions,^5^ their microporosity (≤2.0 nm) is known to be dominated by both kinetic and thermodynamic factors during the crystallization process.^6^ However, it is also true that the phase selectivity of the crystallization is largely altered by the primary chemical composition of synthesis mixtures. Further, changes in the synthesis gel composition can lead to shifts in the chemical equilibrium from the crystallization of metastable zeolitic materials to the formation of non-microporous amorphous and dense phases.^7,8^ Nevertheless, little is known about whether any universal synthesis factor that determines the microporosity, as well as the phase selectivity, of the solid product can exist.

The composition of zeolites and zeolite-like materials can be divided into two main groups, that is, silicate- and phosphate-based molecular sieves. Unlike the former one, the latter group of materials such as aluminophosphate (AlPO_4_), silicoaluminophosphate (SAPO), and metalloalumino-phosphate (MeAPO) molecular sieves^9–11^ are generally synthesized using expensive organic structure-directing agents (OSDAs). Since the OSDAs remain encapsulated within the pore space of the as-synthesized material, they are eliminated by high-temperature calcination that emits environmentally harmful products. Also, this process can often lead to structural collapse, for example, due to enhanced water susceptibility,^12,13^ making the calcined material unviable for applications. Therefore, while many attempts on the OSDA-free synthesis of SAPO molecular sieves have already been made by considering P substitution into the zeolite framework,^14–19^ none of them have clearly shown the isomorphous substitution of this element into the framework tetrahedral sites (T-sites).

On the other hand, we have recently been able to crystallize the SAPO versions of small-pore zeolites analcime (framework type ANA), edingtonite (EDI), gismondine (GIS), and merlinoite (MER), using various alkali metal ions under wholly inorganic conditions and to confirm the presence of P atoms in their T-sites.^20^ However, despite this, the wide utilization of inorganic structure-directing agents (ISDAs) such as alkali and alkaline earth metal ions has not yet begun to make inroads into the synthesis of various phosphate-based molecular sieves including MeAPO materials, due to the limited knowledge of the key parameter that affects the formation of the product structure. Indeed, if such a factor is already present, its discovery would be a step forward in the rational synthesis of new zeolite structures and compositions.

**Fig. 1:**
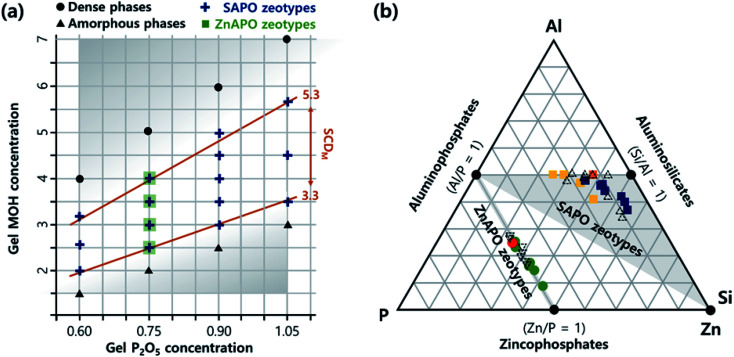
(a) The relation between the MOH and P_2_O_5_ concentrations in SAPO (+) and ZnAPO (▪) synthesis gels, where M is the alkali metal ion, termed the synthesis charge density (SCD_M_) in this study. The crystallization of zeolitic phases was always observed under the conditions *ca.* 3.3 ≤ SCD_M_ ≤ 5.3 when *ca.* 0.6 ≤ gel P_2_O_5_ concentration ≤ 1.1, *i.e.*, within the white domain with SCD_M_ limits indicated by orange lines. The formation of an amorphous or dense phase as the major synthetic product was observed outside of the SCD_M_ range (grey domain). (b) Ternary diagram illustrating the chemical compositions of SAPO (■) and ZnAPO (●) molecular sieves obtained: Na-SAPO-CHA (yellow), Na-SAPO-FAU/EMT (orange), Na-SAPO-LTA (blue), Na-ZnAPO-CZP (green), and Na-ZnAPO-SOD (red). The compositions of a selected series of known SAPO (△; K-SAPO-MER, K-SAPO-EDI, Na-SAPO-GIS, and Cs-SAPO-ANA) materials with Si/(Si + Al + P) = 0.31–0.54 (ref. 20) and ZnAPO (▽; PST-16) (KDEDMA-ZnAPO-CGS), PST-17 (KDEDMA-ZnAPO-BPH), PST-19 (KTEA-ZnAPO-SBS) and ZnAPO-88 (KTEA-ZnAPO-MER) ones with Zn/(Zn + Al + P) = 0.24–0.34,^26^ where DEDMA and TEA are the diethyldimethylammonium and tetraethylammonium ions, respectively, are also given for comparison.

Here we report that an experimentally verified but generalizable synthesis parameter does exist, and that it determines the microporosity and phase selectivity in the synthesis of SAPO and zincoaluminophosphate (ZnAPO) molecular sieves. The gel MOH/P_2_O_5_ ratio in the range *ca.* 3.3–5.3, where M is the alkali metal ion used, governs the ISDA-mediated crystallization of SAPO molecular sieves, while inhibiting the formation of non-zeolitic phases like layered and dense materials. This empirical parameter, named the synthesis charge density (SCD_M_), allowed us to find high-charge-density SAPO molecular sieves with CHA and LTA topologies, and a SAPO FAU/EMT inter-growth using only ISDAs *via* the charge density matching approach.^21,22^ Notably, the SCD_M_ concept, which has never been recognized until now, was also successfully applied to the synthesis of ZnAPO molecular sieves with CZP and SOD topologies without using any ODSAs (Fig. 1), further demonstrating its generalizability.

## 2 Experimental section

### 2.1 Synthesis

The reagents used for the synthesis of SAPO and ZnAPO molecular sieves included NaOH (50 wt% in water, Aldrich), RbOH (50 wt% in water, Aldrich), CsOH (50 wt% in water, Aldrich), aluminium hydroxide (99%, Aldrich), aluminium isopropoxide (99%, Aldrich), phosphoric acid (85%, Aldrich), Ludox® TMA-34 (34% colloidal silica in water, Aldrich), zinc acetate dihydrate (99%, Aldrich), and deionized water. The gel composition for each synthesis was carefully selected with respect to the P_2_O_5_/Al_2_O_3_ ratios of 0.9 and 6.0, respectively. All synthesis gels were prepared by high-speed stirring for homogeneous mixing of the reagents.

In a typical synthesis of SAPO molecular sieves, gels with the composition *x*MOH·1.0Al_2_O_3_·0.9P_2_O_5_·*y*SiO_2_·*z*H_2_O, where M is alkali metal ions and *x* and *y* are varied between 3.00 ≤ *x* ≤ 5.00, 0.50 ≤ *y* ≤ 2.75, and 110 ≤ *z* ≤ 200, respectively, were used. Here, aluminium hydroxide was mixed with phosphoric acid in water, and stirred at room temperature for 6 h. To this slurry, given amounts of Ludox® TMA-34 and an alkali metal hydroxide were successively added dropwise. After being stirred for another 24 h at room temperature, the final synthesis mixture was charged into Teflon-lined 23 mL Parr autoclaves and heated under static conditions at 115–150 °C for up to 2 weeks. The synthesis of SAPO-FAU and SAPO-FAU/EMT was performed using SAPO gels with composition *x*NaOH·*y*ROH·1.0Al_2_O_3_·0.9P_2_O_5_·1.5SiO_2_·150H_2_O, where *x* and *y* are varied between 3.0 ≤ *x* ≤ 4.0 and 0.0 ≤ *y* ≤ 1.0 to make the sum of *x* and *y* 4.0 and R is tetramethylammonium (TMA^+^) or tetraethylammonium (TEA^+^) employed as a replacement of Na^+^. If required, a small amount (4 wt% of the alumina in the gel) of zeolite Na–Y (Si/Al = 2.6, Aldrich) was added as seed crystals. The same synthesis procedure was also adopted to the additional synthesis systems using sodium SAPO gels with the extended P_2_O_5_/Al_2_O_3_ ratios of 0.60 and 1.05. The solid products were recovered by filtration, washed repeatedly with de-ionized water, and then dried overnight at room temperature.

In a typical synthesis of Na-ZnAPO-CZP, gels with composition *x*NaOH·0.750P_2_O_5_·0.125Al_2_O_3_·*y*ZnO·110H_2_O, where *x* and *y* are varied between 2.50 ≤ *x* ≤ 4.00 and 0.25 ≤ *y* ≤ 1.00, respectively, were prepared as follows. Aluminium isopropoxide was mixed with NaOH in water, and stirred at room temperature for 1 h. To this mixture, a given amount of phosphoric acid solution was added, and then stirred for 2 h. Zinc acetate dehydrate was further added to this mixture with agitation for another 1 h. In the case of Na-ZnAPO-SOD synthesis, aluminium isopropoxide was added last to its synthesis gel prepared by stirring for 2 h a mixture where NaOH, zinc acetate dehydrate and phosphoric acid were successively added into water. The crystallization of Na-ZnAPO-CZP and Na-ZnAPO-SOD was carried out under rotation (60 rpm) and statically at 135 °C for 12 h, respectively.

For gas adsorption comparison, Na-chabazite (CHA) with Si/Al = 2.2 was synthesized according to the procedure reported in the literature.^23^ In addition, Na-X (FAU) with Si/Al = 1.3 and Na-A (LTA) with Si/Al = 1.0 were obtained from Tosoh and Aldrich, respectively. Ion-exchange of the NaTMA-SAPO-FAU sample was done by stirring the as-made sample in 0.1 M NaNO_3_ solution at 60 °C for 6 h, and this process was repeated twice.

### 2.2 Analytical methods

The phase purity and crystallinity were determined by PXRD on a PANalytical X'Pert diffractometer (Cu K_α_ radiation) with an X'Celerator detector. *In situ* variable-temperature PXRD experiments were conducted in Bragg–Brentano geometry using the same diffractometer with an Edmund Bühler HDK 1.4 high temperature attachment. Data were collected with a fixed divergence slit (0.50°) and Soller slits (incident and diffracted = 0.04 rad). When necessary, the synchrotron PXRD data were collected at room temperature in flat plate mode on the beamline 5A of the Pohang Acceleration Laboratory (PAL, Pohang, Korea) using a monochromated X-ray (*λ* = 1.51670 Å). The PXRD patterns of the representative SAPO and ZnAPO materials obtained in this work were successfully indexed in their respective unit cells using the DICVOL program implemented in the FULLPROF suite.

Crystal morphology and size were determined with a JEOL JSM-6300 SEM. Elemental analysis was carried out by the analytical laboratory of the Pohang Institute of Metal Industry Advancement. Thermogravimetric and differential thermal analyses (TGA/DTA) were performed in air on a TA Instruments SDT 2960 thermal analyzer at a heating rate of 10 °C min^−1^. TEM maps of SAPO and ZnAPO materials synthesized here were obtained on a JEOL JEM-2200FS TEM at the Jeonju Center of the Korea Basic Science Institute (KBSI). The N_2_ sorption measurements on some X-ray amorphous SAPO or ZnAPO materials were carried out using a Mirae SI nanoPorosity-XG analyzer.

^27^Al, ^29^Si, and ^31^P MAS NMR measurements were carried out on a Bruker DRX500 spectrometer at a spinning rate of 15.0 kHz. If required, the solid sample was dehydrated under a residual pressure of 10^−3^ Torr at 200 or 300 °C for 6 h prior to NMR measurements. ^27^Al MAS NMR spectra were recorded at a ^27^Al frequency of 130.351 MHz with a π/6 rad pulse length of 1.0 μs, a recycle delay of 2.0 s and an acquisition of *ca.* 1000 pulse transients. ^29^Si MAS NMR spectra were recorded at a ^29^Si frequency of 99.362 MHz with a π/2 rad pulse length of 4.0 μs, a recycle delay of 60 s and an acquisition of about 5000 pulse transients. ^31^P MAS NMR spectra were obtained at a ^31^P frequency of 202.457 MHz with a π/6 rad pulse length of 4.0 μs, a recycle delay of 5.0 s and an acquisition of 128 pulse transients. The ^27^Al, ^29^Si, and ^31^P chemical shifts are reported relative to an Al(H_2_O)_6_^3+^ solution, tetramethylsilane and a H_3_PO_4_ solution, respectively.

Gas adsorption experiments were performed using a Mirae SI nanoPorosity-XG analyzer. CO_2_ (99.999%), N_2_ (99.995%), and CH_4_ (99.995%) adsorption isotherms were recorded at 25 °C and pressures up to 1.2 bar. Prior to the experiments, 0.1 g of sample was evacuated under a vacuum of 10^−3^ Torr at 250 °C for 6 h. The temperature of the samples was subsequently reduced under vacuum until the target temperature was reached. The criterion of equilibration at a given pressure point depends on the rate of adsorption, as such, a maximum equilibration time of 30 min was set for each isotherm point.

## 3 Results and discussion

Table 1 lists the representative products obtained after heating sodium-containing SAPO and ZnAPO gels with different chemical compositions at various crystallization temperatures for 3 h to 2 weeks. All molecular sieves synthesized were referred to as Na-SAPO-FTC or Na-ZnAPO-FTC, where FTC is the framework type code of each phase.^24^ Additionally, when an OSDA cation was used in the synthesis, its acronym was added to the name of the crystallized product. We have recently shown a linear relationship between the gel MOH/Al_2_O_3_ or SiO_2_/Al_2_O_3_ ratio and the framework Si fraction in crystallized SAPO molecular sieves.^20^ But here we show that the phase selectivity of the crystallization is actually dependent on the MOH/P_2_O_5_ ratio or SCD_M_ in SAPO (or ZnAPO) gels when the MOH/Al_2_O_3_ and SiO_2_/Al_2_O_3_ (or ZnO/Al_2_O_3_) ratios are chemically feasible.

**Table 1:** SAPO and ZnAPO molecular sieve synthesis conditions and results

Run	Gel composition^a^					
	H	NaOH/P_2_O_5_	(SiO_2_ or ZnO)/P_2_O_5_	H_2_O/Al_2_O_3_	*T*/*t* (°C/h)	Product^b^
1	Si	5.56	1.67	110	150/48	Na-SAPO-LTA
2	Si	5.56	2.22	110	150/48	Na-SAPO-LTA
3	Si	5.56	2.78	110	150/48	Na-SAPO-LTA
4	Si	5.56	3.06	110	150/48	Na-SAPO-LTA
5–7	Si	6.11	1.67–3.33	110	150/24	D + Na-SAPO-LTA
8–10	Si	6.67	1.67–3.33	110	150/12	D
11	Si	5.00	1.67	110	150/48	Na-SAPO-LTA
12	Si	5.00	2.22	110	150/48	Na-SAPO-LTA
13	Si	5.00	2.78	110	150/48	Na-SAPO-LTA
14^c^	Si	4.44	1.67	150	115/216	Na-SAPO-FAU/EMT
15^c^^,^^d^	Si	3.33	1.67	150	115/216	NaTMA-SAPO-FAU
16^c^^,^^d^	Si	3.33	1.67	150	115/216	NaTEA-SAPO-FAU/EMT
17	Si	3.89	1.67	200	150/120	Na-SAPO-CHA
18	Si	3.89	2.22	200	150/120	Na-SAPO-CHA
19	Si	3.33	0.56	200	150/120	Na-SAPO-CHA
20–22	Si	2.78	0.28–1.67	200	150/336	A
23	Zn	5.33	1.33	880	135/12	Na-ZnAPO-CZP
24–26	Zn	6.00	0.33–1.67	880	135/3	D + Na-ZnAPO-CZP
27–29	Zn	6.67	0.33–1.67	880	135/3	D
30	Zn	4.67	0.33	880	135/12	Na-ZnAPO-CZP
31	Zn	4.67	0.67	880	135/12	Na-ZnAPO-CZP
32	Zn	4.00	0.33	880	135/12	Na-ZnAPO-CZP
33	Zn	4.00	0.67	880	135/12	Na-ZnAPO-CZP
34	Zn	3.33	0.33	880	135/12	Na-ZnAPO-CZP
35^e^	Zn	3.33	0.33	880	135/12	Na-ZnAPO-SOD
36–38	Zn	2.67	0.17–0.67	880	135/168	A

a H is the heteroatom (Si or Zn) added to the synthesis gel of each product. Crystallization was performed under static conditions, using SAPO (or ZnAPO) gels with P_2_O_5_/Al_2_O_3_ = 0.90 and 6.00, respectively.

b The product appearing first is the major phase. A and D indicate amorphous and dense (*i.e.*, cristobalite or tridymite) phases, respectively.

c A small amount (4 wt% of the alumina in the gel) of zeolite Na–Y with Si/Al = 2.6 was added as seed crystals.

d TMAOH or TEAOH was added, in addition to NaOH, to achieve a (NaOH + (TMAOH or TEAOH))/P_2_O_5_ ratio of 4.44.

e The Al source was added last to the gel, unlike the other ZnAPO gels where the Zn source was added last.

Given the competitive incorporation of Si and P atoms into the AlPO_4_ framework during the crystallization process,^11^ we first selected P_2_O_5_/Al_2_O_3_ and SiO_2_/P_2_O_5_ ratios of 0.90 and 1.67, which are *ca.* 20% increments of those (0.75 and 1.33, respectively) used in our recent paper,^20^ in the sodium SAPO gel. In addition, an increase of *ca.* 40% H_2_O content (H_2_O/Al_2_O_3_ = 110) was also adopted to prepare a homogeneous synthesis mixture. When the NaOH/P_2_O_5_ ratio (SCD_Na_) ranges from 5.00 to 5.56 in this synthesis mixture, thereby making it more siliceous (1.67 ≤ SiO_2_/P_2_O_5_ ≤ 3.06) and more basic (11.4 ≤ pH ≤ 12.8; Table S1, ESI†), we were able to obtain a series of Na-SAPO-LTA materials with different Si contents after heating at 150 °C for 48 h.

On the other hand, the synthesis using gels with lower SCD_Na_ values (3.33–3.89) and lower Si contents (0.56 ≤ SiO_2_/P_2_O_5_ ≤ 2.22) at 150 °C for 5 days yielded Na-SAPO-CHA (Table 1). We also found that the use of a small amount (2 wt% of the alumina in the gel) of Na-SAPO-CHA, obtained from run 17 in Table 1, as seeds inhibits the formation of Na-SAPO-GIS that frequently co-crystallized with Na-SAPO-CHA at SiO_2_/P_2_O_5_ ratios below 1.67. This stimulated us to further investigate the seeding effect under the ISDA-mediated synthesis conditions of pre-established SCD_Na_ values (3.33–5.56) at different temperatures. While a FAU-like material was observed as the minor phase in the product obtained after heating a SAPO gel with SCD_Na_ = 4.44 and SiO_2_/P_2_O_5_ = 1.67 at a lower temperature (115 °C) for 9 days, no noticeable changes in the phase selectivity were caused by adding 4 wt% organically synthesized SAPO-37 (FAU) with Si/(Si + Al + P) = 0.13.

However, the use of the same amount of zeolite Na–Y with Si/Al = 2.6 as seeds gave Na-SAPO-FAU/EMT, an intergrowth of Na-SAPO-FAU and Na-SAPO-EMT materials. Therefore, since no seed crystals were found to be detectable after aging of the SAPO synthesis gel at room-temperature for 24 h (Fig. S1, ESI†), the framework charge density (FCD; the number of charged heteroatoms over that of total framework atoms, *i.e.*, the (Al–P)/(Si + Al + P) and Al/(Si + Al) ratios for SAPO molecular sieves and aluminosilicate zeolites, respectively) of seed crystals appears to be more important than the type of framework elements in the OSDA-free synthesis of SAPO molecular sieves. Table 1 also shows that the replacement of one fourth of the NaOH in the synthesis mixture containing Na–Y seeds for Na-SAPO-FAU/EMT formation by tetraethylammonium (TEA^+^) hydroxide led to NaTEA-SAPO-FAU/EMT, whereas the use of the smaller tetramethylammonium (TMA^+^) ion resulted in pure NaTMA-SAPO-FAU (Fig. S2, ESI†). To date, the synthesis of a pure SAPO-FAU material (*i.e.*, SAPO-37) has only been possible in the presence of tetrapropylammonium (TPA^+^) and TMA^+^ ions at a tightly controlled TPA^+^/TMA^+^ ratio of 1 : 40 in the gel.^10,25^ This again shows that the combined use of ISDAs and OSDAs is a viable alternative toward the synthesis of SAPO molecular sieves with novel framework compositions.^26^

Another interesting observation from Table 1 is that while the SAPO gels with SCD_Na_ < 3.3 remain mainly amorphous with no microporosity (Table S2, ESI†) even after two weeks of heating at different temperatures (115–150 °C), regardless of their SiO_2_/P_2_O_5_ ratios, the formation of dense phases such as cristobalite is always inevitable at SCD_Na_ > 5.6 from the beginning of the synthesis. The same trend was observed even when the gel pH was adjusted to 12.5 (or 8.5), where the crystallization of Na-SAPO-LTA (or Na-SAPO-CHA) was successful, by adding a nominal amount of HNO_3_ (or TMAOH or TEAOH) (Tables S1 and S3, ESI†). Therefore, we considered the possibility that SCD_M_ could be an intrinsic parameter determining the microporosity of solid products in the synthesis of SAPO zeotypes, independently of the pH of their gels, as well as the phase selectivity of the crystallization.

Indeed, combining all results from the Na^+^-mediated synthesis of SAPO molecular sieves at P_2_O_5_/Al_2_O_3_ = 0.90 with those of our recent K^+^-mediated ones at P_2_O_5_/Al_2_O_3_ ratio = 0.75 (ref. 20) reveals that zeolitic phase crystallization safely occurs within the SCD_M_ range *ca.* 3.3–5.3. More importantly, further deceasing or increasing the gel P_2_O_5_/Al_2_O_3_ ratio to 0.60 or to 1.05, 20% lower or higher than the two low (0.75) or high (0.90) P_2_O_5_/Al_2_O_3_ ratios described above, respectively, again directed the synthesis of SAPO molecular sieves at *ca.* 3.3 ≤ SCD_M_ ≤ 5.3 (Table S4, ESI† and Fig. 1a). It is also worth noting that the use of other alkali cations, *i.e.*, Rb^+^ and Cs^+^, led to SAPO materials like Rb-SAPO-MER and Cs-SAPO-ANA in the same SCD_M_ region when the P_2_O_5_/Al_2_O_3_ ratio is 0.90 (Table S5, ESI†). These results clearly show that the SCD_M_ concept, characterized by the specific range of gel MOH/P_2_O_5_ ratios, is more crucial than the other important synthesis parameters (*i.e.*, gel P_2_O_5_/Al_2_O_3_ and SiO_2_/P_2_O_5_ ratios) in the ISDA-mediated synthesis of SAPO molecular sieves, because the latter two parameters can differ notably according to the framework composition of the crystallized product.

To examine whether this unprecedented parameter is generalizable to the synthesis of other families of phosphate-based molecular sieves, we applied it to the ZnAPO composition system, as a representative case study of the OSDA-free synthesis of MeAPO molecular sieves which has not been reported yet. Unlike Si substitution into the AlPO_4_ framework, divalent Zn cannot substitute for P,^11^ so that any ZnAPO phase crystallized has no ability to self-tune its charge density with respect to the total charge density of the extra-framework species employed. Therefore, the amount of Zn in ZnAPO gels was adjusted to be lower than or equal to the difference between the P and Al amounts, but higher than or equal to the Al one, *i.e.*, Al ≤ Zn ≤ (P–Al). We also adopted an excess phosphate gel system (P/(Al + Zn) ≥ 2.0) to ensure that framework element components are soluble.^26^

Using sodium ZnAPO gels with oxide compositions described above, we were able to obtain Na-ZnAPO-CZP, a chiral large-pore zeolite with one-dimensional 12-membered ring channels originally synthesized as ZnPO_4_ and CoZnPO_4_ compositions in the presence of Na^+^ as an ISDA,^27^ after 12 h of heating at 135 °C. It should be noted that the SCD_Na_ range leading to this ZnAPO material is exactly the same as that observed for the ISDA-mediated synthesis of SAPO molecular sieves (Tables 1, S4, and S5, ESI†). When the SCD_Na_ was lower than 3.3 and higher than 5.3, on the other hand, non-microporous amorphous (Table S2, ESI†) and dense (*i.e.*, tridymite) materials were the main products obtained after 1 week and 3 h of heating at the same crystallization temperature, respectively. On the basis of results presented here, therefore, we conclude that despite its empirical nature, SCD_M_ is a simple and reliable parameter governing the microporosity and phase selectivity in the ISDA-mediated synthesis of zeolite-like materials with charged frameworks such as SAPO and ZnAPO molecular sieves. The SCD_M_ concept can be rationalized by considering zeolite crystallization based on ISDA-framework charge density matching. It is not difficult to infer that SCD_M_ values within a particular range (*ca.* 3.3 ≤ SCD_M_ ≤ 5.3) can optimize the solubility of inorganic framework species, as well as the charge balancing between them and ISDAs, leading to their successful incorporation into the framework of the crystallized product.

It is also remarkable that Na-ZnAPO-CZP was always obtained using synthesis gels where the Zn source was added last. However, when the Al source was the final component added, Na-ZnAPO-SOD was the phase formed not only from a gel with SCD_Na_ = 3.33 and ZnO/P_2_O_5_ = 0.33, but also from gels with higher SCD_Na_ values up to 5.33, although not phase-pure (Table S6, ESI†). This change in phase selectivity can be rationalized by considering the difference in host lattices (AlPO_4_ framework *vs.* ZnPO_4_ one) for heteroatom (Zn *vs.* Al) substitution.

**Fig. 2:**
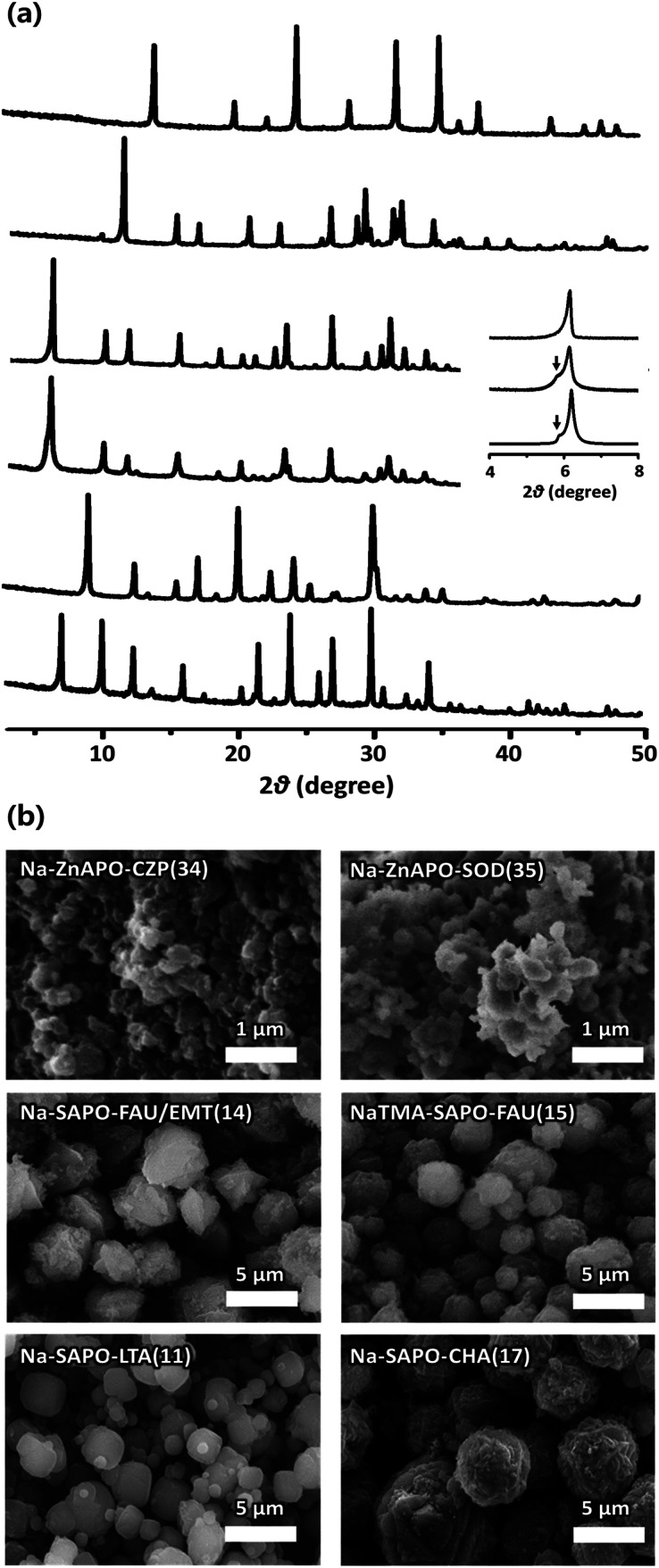
(a) PXRD patterns and (b) SEM images of as-synthesized (from bottom to top) Na-SAPO-LTA(11), Na-SAPO-CHA(17), Na-SAPO-FAU/EMT(14), NaTMA-SAPO-FAU(15), Na-ZnAPO-CZP(34), and Na-ZnAPO-SOD(35) where TMA is the tetramethylammonium ion. The numbers in parentheses attached to the material name are the same as the synthesis run numbers in Table 1. The inset in (a) compares the experimental PXRD patterns (top two traces) in the 2*θ* region 4–8° of Na-SAPO-FAU/EMT(14) and NaTMA-SAPO-FAU(15) with the simulated one (bottom trace) of a FAU/EMT intergrowth structure at a ratio of 80 : 20.^24,28^ The arrow indicates the (100) reflection of the EMT structure.

The powder X-ray diffraction (PXRD) patterns of the representative SAPO and ZnAPO molecular sieves synthesized here show that they are highly crystalline and no reflections other than those of each corresponding zeolite structure are observed (Fig. 2a).^24^ An interesting observation is that the PXRD pattern of Na-SAPO-FAU/EMT(14), where the number in parentheses is the run number in Table 1, is characterized by a shoulder around 2*θ* = 5.8°, as well as by a sharp reflection at 2*θ* = 6.2°. A careful comparison with the PXRD data of the FAU/EMT intergrowth family^24,28^ reveals that this SAPO material is an intergrowth with a proportion of the FAU phase around 80%. To our knowledge, Na-SAPO-FAU/EMT(14) is the first example where the EMT structure has been realized as the SAPO composition. The scanning electron microscopy (SEM) images in Fig. 2b show that SAPO and ZnAPO molecular sieves synthesized here possess uniform morphologies. This is also true for Na-SAPO-FAU/EMT(14) that typically appears as spherical aggregates of approximately 5 μm, which in turn consist of heavily overlapped octahedra with an average size of *ca.* 2 μm.

**Fig. 3:**
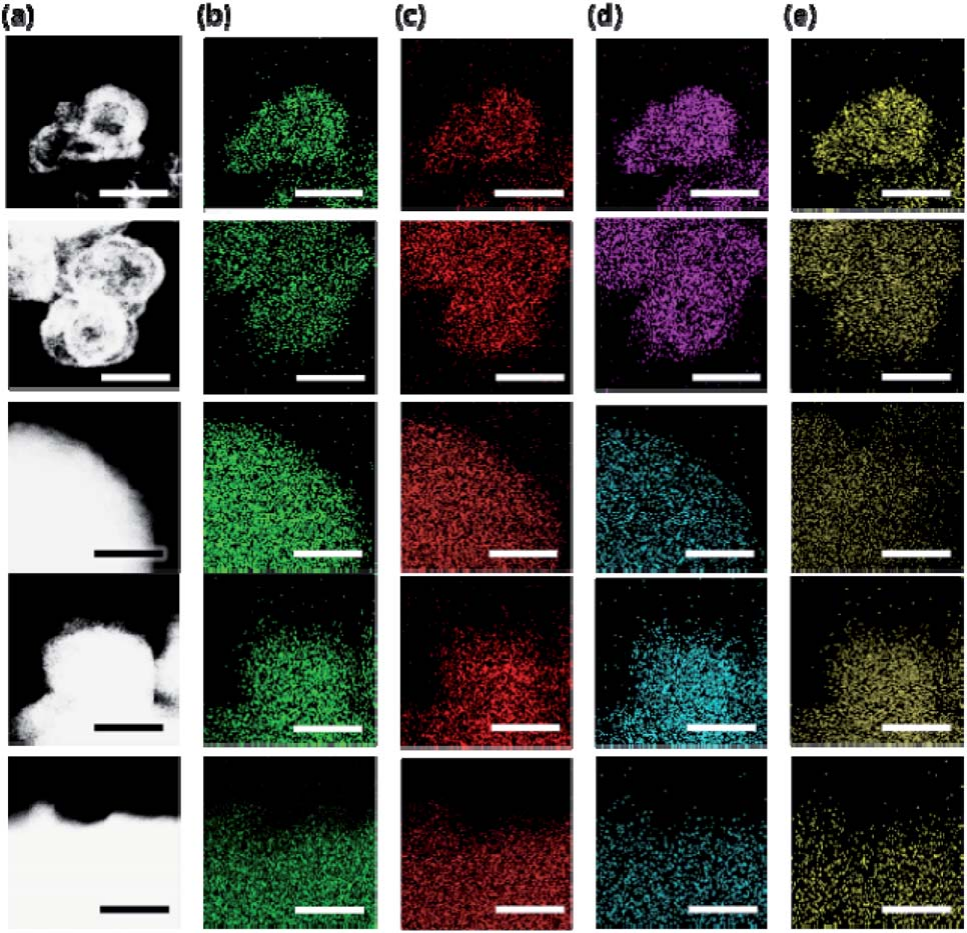
(a) TEM and (b) Al, (c) P, (d) Si (turquoise) or Zn (pink), and (e) Na TEM elemental mapping images of the as-synthesized (from bottom to top) Na-SAPO-LTA(11), Na-SAPO-CHA(17), Na-SAPO-FAU/EMT(14), Na-ZnAPO-CZP(34), and Na-ZnAPO-SOD(35). Scale bars: 200 nm.

Elemental analysis indicates that the Si (or Zn) incorporation in our SAPO (or ZnAPO) molecular sieves always exceeds that of P (or Al), whereas the Al (or P) level is close to half the tetrahedral atoms (T-atoms) per unit cell of each material (Fig. 1b and Table 2). Consequently, all SAPO materials have unusually high framework Si fractions (0.25 ≤ Si/(Si + Al + P) ≤ 0.55) and FCD values (0.25–0.38) due to the use of high-charge-density ISDAs in their synthesis.^20^ Here, we note that NaTMA-SAPO-FAU(15) has a FCD value of 0.38, significantly higher than that (0.13) of SAPO-37.^10,25^ However, the FCD of our SAPO materials was found to be not always proportional to the framework Si fraction (Table 2), due to the Si substitution for Al–P pairs, as well as for P atoms, leading to the formation of neutral Si islands during the crystallization.^10^ On the other hand, since Zn in the series of Na-ZnAPO-CZP materials can substitute for Al only,^11^ a linear relationship between the FCD and framework Zn fraction was observed. Table 2 also shows that the FCD (0.28) of Na-ZnAPO-CZP(34) is essentially identical to that (0.27) of Na-ZnAPO-SOD(35), when synthesized using gels with the same SCD_Na_ = 3.33 and ZnO/P_2_O_5_ = 0.33. Transmission electron microscopy (TEM) mapping clearly shows the homogeneous distributions of both the framework elements (Al, P, and Si or Zn) and the extra-framework alkali cation (Na^+^) in our SAPO and ZnAPO materials (Fig. 3).

**Table 2:** Unit cell compositions and parameters of SAPO and ZnAPO molecular sieves synthesized in this work

Material	Unit cell composition^a^	H/(H + Al + P)^b^	|Al–P|/(H + Al + P)^c^	Unit cell parameters (Å) and volume (Å^3^)^d^
Na-SAPO-LTA(1)	|Na_9.5_(OH)_9.9_(H_2_O)_23.8_|[Al_11.2_P_2.6_Si_10.1_O_48_]	0.42	0.36	*a* = 24.617(8), *V* = 14 917.8(2)
Na-SAPO-LTA(2)	|Na_9.7_(OH)_1.5_(H_2_O)_23.7_|[Al_10.0_P_1.8_Si_12.2_O_48_]	0.46	0.35	
Na-SAPO-LTA(3)	|Na_8.5_(OH)_0.9_(H_2_O)_24.0_|[Al_9.5_P_1.9_Si_12.6_O_48_]	0.53	0.32	
Na-SAPO-LTA(4)	|Na_8.6_(OH)_1.5_(H_2_O)_23.8_|[Al_9.0_P_1.9_Si_13.1_O_48_]	0.55	0.29	
Na-SAPO-LTA(11)	|Na_7.9_(OH)_0.2_(H_2_O)_24.7_|[Al_11.5_P_3.8_Si_8.7_O_48_]	0.36	0.32	
Na-SAPO-LTA(12)	|Na_9.6_(OH)_1.4_(H_2_O)_23.6_|[Al_11.0_P_2.8_Si_10.2_O_48_]	0.43	0.34	
Na-SAPO-LTA(13)	|Na_7.6_(OH)_0.4_(H_2_O)_24.8_|[Al_10.3_P_3.1_Si_11.2_O_48_]	0.46	0.29	*a* = 24.610(5), *V* = 14 905.0(9)
Na-SAPO-FAU/EMT(14)	|Na_71.0_(OH)_0.1_(H_2_O)_233.9_|[Al_95.6_P_24.7_Si_71.6_O_384_]	0.37	0.37	
NaTMA-SAPO-FAU(15)	|Na_42.8_TMA_28.9_(H_2_O)_110.9_|[Al_95.8_P_24.1_Si_72.1_O_384_]	0.38	0.37	*a* = 24.971(7), *V* = 15 570.6(8)
NaTEA-SAPO-FAU/EMT(16)	|Na_66.7_TEA_7.2_(H_2_O)_197.5_|[Al_97.9_P_24.0_Si_71.0_O_384_]	0.37	0.38	
Na-SAPO-CHA(17)	|Na_11.6_(OH)_1.4_(H_2_O)_35.1_|[Al_16.8_P_6.6_Si_12.7_O_72_]	0.35	0.28	*a* = 13.918(7), *c* = 15.260(5), *V* = 2956.4(6)
Na-SAPO-CHA(18)	|Na_9.8_(OH)_1.0_(H_2_O)_33.7_|[Al_14.9_P_6.1_Si_15.0_O_72_]	0.42	0.25	
Na-SAPO-CHA(19)	|Na_9.1_(OH)_0.6_(H_2_O)_36.4_|[Al_17.7_P_9.2_Si_9.1_O_72_]	0.25	0.25	*a* = 13.910(3), *c* = 15.255(5), *V* = 2951.7(8)
Na-ZnAPO-CZP(23)	|Na_11.5_(OH)_1.4_(H_2_O)_14.9_|[Al_2.1_P_11.7_Zn_10.3_O_48_]	0.40	0.43	*a* = 8.998(5), *V* = 728.5(1)
Na-ZnAPO-CZP(30)	|Na_9.4_(OH)_1.7_(H_2_O)_16.1_|[Al_4.0_P_11.7_Zn_8.3_O_48_]	0.32	0.35	
Na-ZnAPO-CZP(31)	|Na_10.2_(OH)_2.0_(H_2_O)_15.8_|[Al_3.6_P_11.8_Zn_8.5_O_48_]	0.34	0.36	
Na-ZnAPO-CZP(32)	|Na_7.1_(OH)_1.1_(H_2_O)_18.5_|[Al_5.9_P_11.9_Zn_6.2_O_48_]	0.32	0.32	
Na-ZnAPO-CZP(33)	|Na_8.3_(OH)_0.3_(H_2_O)_17.6_|[Al_4.0_P_12.0_Zn_8.5_O_48_]	0.33	0.34	
Na-ZnAPO-CZP(34)	|Na_7.6_(OH)_1.2_(H_2_O)_18.3_|[Al_5.4_P_12.2_Zn_6.4_O_48_]	0.27	0.28	*a* = 8.882(8), *V* = 700.7(0)
Na-ZnAPO-SOD(35)	|Na_4.9_(OH)_1.7_(H_2_O)_10.3_|[Al_2.8_P_6.0_Zn_3.2_O_24_]	0.27	0.27	*a* = 10.536(4), *V* = 1169.5(7)

a Determined from a combination of elemental and thermal analyses. The water content was calculated from the endothermic weight loss by thermal analysis up to 200 °C, and OH has been introduced to make as-synthesized molecular sieves electrically neutral.

b Framework heteroatom (H = Si or Zn) fraction.

c Framework charge density.

d Determined using low-resolution PXRD data.

**Fig. 4:**
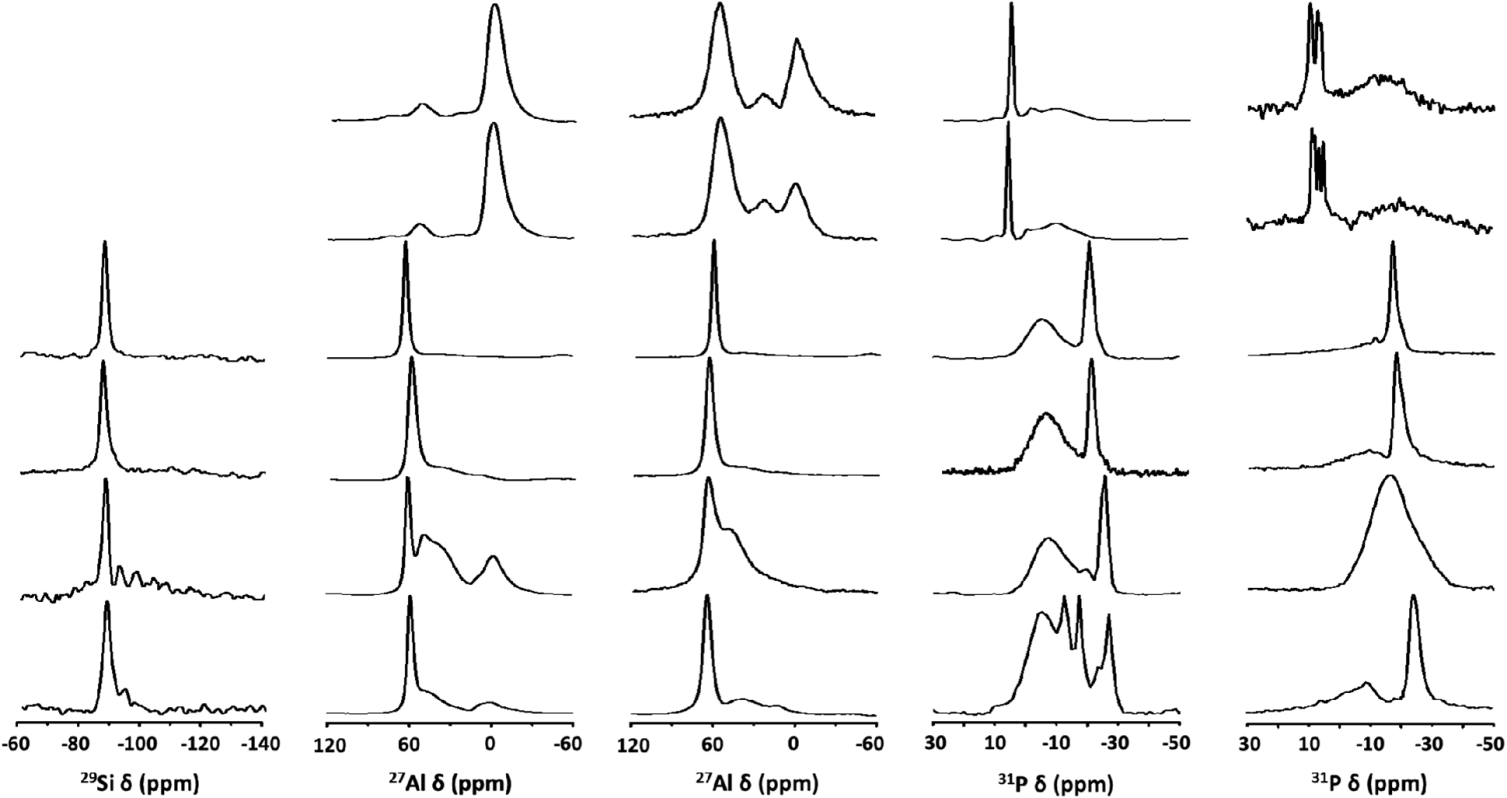
(a) ^29^Si, (b and c) ^27^Al, and (d and e) ^31^P MAS NMR spectra of as-synthesized (from bottom to top) Na-SAPO-LTA(11), Na-SAPO-CHA(17), Na-SAPO-FAU/EMT(14), NaTMA-SAPO-FAU(15), Na-ZnAPO-CZP(34), and Na-ZnAPO-SOD(35), where TMA is the tetramethylammonium ion, in their hydrated (a, b, and d) and (partially) dehydrated (c and e) forms. While the SAPO molecular sieves were dehydrated at 300 °C under a residual pressure of 10^−3^ Torr, the ZnAPO materials were partially dehydrated at 200 °C under the same residual pressure to maintain their structural integrity.

^29^Si magic-angle spinning (MAS) nuclear magnetic resonance (NMR) spectroscopy reveals that as-synthesized (hydrated) NaTMA-SAPO-FAU(15) exhibits only one resonance around −90 ppm due to Si(4Al) species, as normally observed for the SAPO-37 materials synthesized using OSDAs only.^10,25^ As seen in Fig. 4a, however, the ^29^Si MAS NMR spectra of Na-SAPO-LTA(11), Na-SAPO-CHA(17), and Na-SAPO-FAU/EMT(14), with quite similar framework Si fractions (0.35–0.37; Table 2), also exhibit weak resonances at lower chemical shifts. This indicates the presence of Si(*n*Al) environments, where *n* = 3–0, due to the Si substitution not only for P but also for Al–P pairs.^29^ The fact that their relative intensities are higher for Na-SAPO-CHA(17) suggests that this material has a higher degree of Si substitution for Al–P pairs than the other two materials, as supported by notable differences in their framework (Si + P)/Al ratios (1.01–1.15).

A similar conclusion can be obtained from the ^27^Al MAS NMR spectra of these three SAPO molecular sieves characterized by different line shapes. For example, the resonance around 40 ppm corresponding to framework Al atoms in P-rich environments^30^ is more clearly visible for hydrated Na-SAPO-CHA(17). However, this is not the case of the spectra of hydrated Na-SAPO-LTA(11) and Na-SAPO-FAU/EMT(14) with more homogeneously distributed framework Si atoms (Fig. 4b). Unlike the SAPO materials that show a prominent ^27^Al resonance around 60 ppm assigned to framework Al atoms in Si-rich environments, on the other hand, hydrated Na-ZnAPO-CZP(34) and Na-ZnAPO-SOD(35) give a weak ^27^Al signal at an upfield chemical shift (*ca.* 50 ppm). The existence of this Al(4P) signal becomes more apparent after partial dehydration under a residual pressure of 10^−2^ Torr at 200 °C (Fig. 4c and S3, ESI†), where their structures remain intact, because of the partial loss of water molecules coordinated to the octahedral framework Al species^31^ in these ZnAPO phases, thus leading to tetrahedral Al framework atoms.

The ^31^P MAS NMR spectra of all SAPO molecular sieves, except Na-SAPO-LTA(11), are characterized by a broad resonance around −5 ppm, as well as by a sharper one appearing between −20 and −30 ppm due to the framework P(4Al) species^20,25^ (Fig. 4d), where the downfield resonance becomes significantly weaker upon dehydration. The appearance of a very broad ^31^P resonance around −18 ppm in the spectrum of dehydrated Na-SAPO-CHA(17) can be due to a structural change of this SAPO material upon loosing water (Fig. S4, ESI†). We also note that the ^31^P MAS NMR spectrum of hydrated Na-SAPO-LTA(11) shows additional resonances between −10 and −20 ppm, assignable to P((4-*n*)Al, *n*OH) species, where *n* is 3, 2, and 1, respectively,^20,32,33^ revealing its highly defective nature, unlike the other SAPO materials prepared here (Fig. 4e). As expected, these resonances diminish after dehydration at 300 °C. Fig. 4 also shows that the ^31^P MAS NMR spectra of hydrated Na-ZnAPO-CZP(34) and Na-ZnAPO-SOD(35) both are characterized by a sharp resonance around 8 ppm, assignable to framework P species tetrahedrally coordinated to Zn as well as Al atoms, which explains the splitting into several resonances after partial dehydration at 300 °C.

No SAPO molecular sieves synthesized here maintained the structural integrity after NH_4_^+^ exchange and subsequent calcination at 550 °C, probably because of the high FCD values.^20^ However, since their Na^+^-form remains intact at this temperature, we examined the CO_2_ adsorption properties at 25 °C of selected samples with various FCD values (Table S7 and Fig. S5, ESI†). As expected, large-pore Na-SAPO-FAU/EMT(14) and NaTMA-SAPO-FAU(15) showed the largest CO_2_ uptake (4.6 mmol g^−1^ at 1.0 bar) among them. However, this value was lower than that (5.8 mmol g^−1^) of Na-X (FAU) with Si/Al = 1.3, mainly due to their lower FCD values (0.37 *vs.* 0.43; Table 2). While a similar trend was observed for Na-SAPO-LTA and Na-SAPO-CHA, it is interesting to note that the CO_2_/CH_4_ and CO_2_/N_2_ selectivities (17 and 36, respectively) of Na-SAPO-CHA(17) with FCD = 0.28 are considerably higher than those (3 and 5, respectively) of Na-chabazite (CHA) with a similar FCD value (0.31; Si/Al = 2.2). Therefore, fine tuning of pore size in SAPO molecular sieves by framework composition control for specific separation applications appears to be possible, like the case of aluminosilicate zeolites.

## 4 Conclusions

In summary, we have demonstrated that an empirical parameter (the MOH/P_2_O_5_ ratio in SAPO and ZnAPO synthesis gels) governs the phase selectivity and microporosity of solid products, as it can distinguish feasible synthesis domains in the presence of alkali metal (M) ions as an ISDA for microporous phosphate-based materials from those leading to the formation of non-zeolitic ones. This simple parameter, designated as the synthesis charge density (SCD_M_), allowed us to synthesize four high-charge-density zeotypes with different framework topologies, as well as a SAPO FAU/EMT intergrowth, when it lies within the range *ca.* 3.3–5.3. However, using synthesis gels with SCD_M_ values outside this range, we always obtained non-zeolitic products, *i.e.*, non-microporous amorphous phases (<3.3) and crystalline but dense ones (>5.3). Intuitively, such an intrinsic parameter should exist in the synthesis of zeolites and molecular sieves. To our knowledge, nevertheless, such a parameter has never been systematically considered in the synthesis of phosphate-based molecular sieves. We anticipate that combining the synthesis charge density concept with the currently available zeolite synthesis database might pave the way for the rational discovery of zeolites and zeotypes with interesting applications in the fields of energy and environment.

## Author contributions

Sung Hwan Park: Conceptualization, Methodology, Formal analysis, Investigation. Kingsley Christian Kemp: Formal analysis, Investigation. Jingeon Hong: Validation. Jung Gi Min: Investigation. Suk Bong Hong: Conceptualization, Supervision. Sung Hwan Park, Kingsley Christian Kemp, and Suk Bong Hong co-wrote the paper: Writing – Original Draft – Review & Editing.

## Conflicts of interest

There are no conflicts to declare.

## Supplementary Material

SC-012-D1SC02431K-s001
