# Supplementary material for: An intrinsic synthesis parameter governing the crystallization of silico(zinco)aluminophosphate molecular sieves
Source: Chem Sci. 2021 Jun 30;12(30):10371–9. doi: 10.1039/d1sc02431k (PMC8336471; doi:10.1039/d1sc02431k)
Supplement: SC-012-D1SC02431K-s001 [file SC-012-D1SC02431K-s001.pdf]

Electronic Supplementary Material (ESI) for Chemical Science.
This journal is © The Royal Society of Chemistry 2021

Electronic Supporting Information

for

An Intrinsic Synthesis Parameter Governing the Crystallization of Silico(zinco)aluminophosphate Molecular Sieves

Sung Hwan Park, Kingsley Christian Kemp, Jingeon Hong, Jung Gi Min, and Suk Bong Hong*

Center for Ordered Nanoporous Materials Synthesis, Division of Environmental Science and Engineering, POSTECH, Pohang 37673, Korea

Table S1. Changes in the pH of the representative SAPO and ZnAPO synthesis mixtures before and after molecular sieve crystallization

run[a]	pH		product[b]
	initial	final	
1	12.6	12.1	Na-SAPO-LTA
2	12.3	11.9	Na-SAPO-LTA
3	12.5	12.3	Na-SAPO-LTA
4	12.8	12.6	Na-SAPO-LTA
11	11.4	11.1	Na-SAPO-LTA
12	11.6	11.4	Na-SAPO-LTA
13	11.6	11.5	Na-SAPO-LTA
14	10.3	9.9	Na-SAPO-FAU/EMT
15	10.3	10.3	NaTMA-SAPO-FAU
16	10.6	10.5	NaTEA-SAPO-FAU/EMT
17	9.3	10.0	Na-SAPO-CHA
18	9.5	9.8	Na-SAPO-CHA
19	8.4	9.0	Na-SAPO-CHA
23	10.4	10.9	Na-ZnAPO-CZP
30	10.5	10.7	Na-ZnAPO-CZP
31	9.6	10.2	Na-ZnAPO-CZP
32	8.3	8.4	Na-ZnAPO-CZP
33	7.3	7.5	Na-ZnAPO-CZP
34	7.3	7.4	Na-ZnAPO-CZP
35	7.4	7.3	Na-ZnAPO-SOD

[a]The same as the run number in Table 1. [b]TMA, tetramethylammonium; TEA, tetraethylammonium.

Table S2. N_2 sorption data for a series of SAPO or ZnAPO amorphous materials.

sample	run[a]	BET surface area (m^2 g^{-1})	Micropore volume (cm^3 g^{-1})[b]	Mesopore volume (cm^3 g^{-1})[c]
SAPO amorphous	20	19	0	0.07
SAPO amorphous	22	55	0	0.25
ZnAPO amorphous	36	35	0	0.17
ZnAPO amorphous	38	33	0	0.15

[a]The same as the synthesis run numbers in the main text Table 1. [b]In the diameter range ≤ 20 Å. [c]In the diameter range 20- to 500-Å. Calculated using the BJH formalism.

Table S3. pH-adjusted SAPO molecular sieve synthesis conditions and results

run	$NaOH/P_2O_5$	SiO_2/P_2O_5	H_2O/Al_2O_3	T/t (°C/h)	pH		product[a]
					initial	final	
39	6.11	1.67	110	150/24	13.5	13.4	D + Na-SAPO-LTA
40[b]	6.11	1.67	110	150/24	12.5	12.9	D + Na-SAPO-LTA
41	6.11	3.06	110	150/24	13.7	13.3	D + Na-SAPO-LTA
42[b]	6.11	3.06	110	150/24	12.5	12.6	D + Na-SAPO-LTA
43	2.78	0.28	200	150/336	7.6	8.4	A
44[b]	2.78	0.28	200	150/336	8.5	8.9	A
45	2.78	1.67	200	150/336	7.8	8.5	A
46[b]	2.78	1.67	200	150/336	8.5	9.0	A

[a]The product appearing first is the major phase. A and D indicate amorphous and dense (i.e., cristobalite) phases, respectively. [b]The initial pH of synthesis gels with different compositions, where the P_2O_5/Al_2O_3 ratio was fixed to 0.9, was adjusted to 12.5 (or 8.5) by adding a small amount of HNO_3 (or TMAOH or TEAOH)).

Table S4. Representative SAPO molecular sieve synthesis conditions and results using gels with an extended P_2O_5/Al_2O_3 ratio of 0.60 or 1.05

run	gel composition[a]				t (h)	product[b]
	P_2O_5/Al_2O_3	$NaOH/P_2O_5$	SiO_2/P_2O_5	H_2O/Al_2O_3		
47	0.60	6.66	1.25	110	24	D
48	0.60	5.83	1.25	110	24	D + Na-SAPO-SOD
49	0.60	5.33	1.25	110	216	Na-SAPO-SOD + Na-SAPO-CHA
50	0.60	4.33	1.25	110	120	Na-SAPO-CHA + Na-SAPO-SOD
51	0.60	3.33	1.25	110	120	Na-SAPO-CHA
52	0.60	2.50	1.25	110	336	A + (Na-SAPO-CHA)
53	0.60	2.78	1.25	110	336	A
54	1.05	6.66	1.90	110	12	D
55	1.05	5.71	1.90	110	12	D + Na-SAPO-SOD
56	1.05	5.33	1.90	110	48	Na-SAPO-SOD + Na-SAPO-LTA
57	1.05	4.76	1.90	110	48	Na-SAPO-LTA
58	1.05	3.33	1.90	110	120	Na-SAPO-CHA + Na-SAPO-GIS
59	1.05	2.86	1.90	110	336	A + (Na-SAPO-GIS)
60	1.05	2.38	1.90	110	336	A

[a]Crystallization was performed at 150 °C under static conditions. [b]The product appearing first is the major phase, and that obtained in a trace amount is given in parentheses. A and D indicate amorphous and dense (i.e., cristobalite) phases, respectively.

Table S5. Representative SAPO molecular sieve synthesis conditions and results using Rb^+ or Cs^+ ions as an ISDA

run	gel composition[a]				t (h)	product[b]
	M	MOH/P_2O_5	SiO_2/P_2O_5	H_2O/Al_2O_3		
61	Rb^+	6.11	1.25	110	24	D
62	Rb^+	5.56	1.25	110	120	Rb-SAPO-MER + (D)
63	Rb^+	4.45	1.25	110	120	Rb-SAPO-MER
64	Rb^+	3.33	1.25	110	168	Rb-SAPO-MER
65	Rb^+	2.78	1.25	110	120	A
66	Cs^+	6.11	1.25	110	336	D + (Cs-SAPO-ANA)
67	Cs^+	5.56	1.25	110	336	Cs-SAPO-ANA + (D)
68	Cs^+	4.33	1.90	110	120	Cs-SAPO-ANA
69	Cs^+	3.33	1.90	110	168	Cs-SAPO-ANA
70	Cs^+	2.78	1.90	110	336	A + (Cs-SAPO-ANA)

[a]Crystallization was performed at 150 °C under rotation (60 rpm), using SAPO gels with P_2O_5/Al_2O_3 = 0.90. [b]The product appearing first is the major phase, and that obtained in a trace amount is given in parentheses. A and D indicate amorphous and dense (i.e., cristobalite) phases, respectively.

Table S6. Syntheses from gel composition xNaOH·0.750P_2O_5·0.125Al_2O_3·yZnO·110H_2O[a]

run	gel composition		t (h)	product[b]
	NaOH/P_2O_5	ZnO/P_2O_5		
71	6.67	1.33	3	D
72	6.00	1.33	3	D + Na-ZnAPO-CZP
73	5.33	1.33	12	Na-ZnAPO-CZP + Na-ZnAPO-SOD
74	4.00	0.33	12	Na-ZnAPO-SOD + Na-ZnAPO-CZP
75	3.33	0.33	12	Na-ZnAPO-SOD
76	2.67	0.33	168	A

[a] x and y are varied between $2.00 \leq x \leq 5.00$ and $0.25 \leq y \leq 1.00$. Crystallization was performed at 135 °C under rotation (60 rpm). [b] The product appearing first is the major phase. A and D indicate amorphous and dense (i.e., tridymite) phases, respectively.

Table S7. Room-temperature CO_2 adsorption data of the SAPO molecular sieves synthesized here.

Material	Framework charge density[b]	CO_2 uptake at 1 bar (mmolg^{-1})	Selectivity[a]	
			CO_2/CH_4	CO_2/N_2
Na-SAPO-LTA(1)	0.36	3.8	7	12
Na-SAPO-LTA(4)	0.29	3.6	6	10
Na-SAPO-CHA(17)	0.28	3.6	17	36
Na-SAPO-CHA(19)	0.25	3.3	9	13
Na-SAPO-FAU/EMT(14)	0.37	4.6	7	13
NaTMA-SAPO-FAU(15)	0.37	4.6	7	12
Na-A (Si/Al = 1.0)	0.50	3.5	5	8
Na-chabazite (Si/Al = 2.2)	0.31	4.3	3	5
Na-X (Si/Al = 1.3)	0.43	5.8	6	9

[a]Defined as Q_{CO2}/Q_{CH4} and Q_{CO2}/Q_{N2}, where Q_{CO2}, Q_{CH4}, and Q_{N2} are the equilibrium molar uptakes of CO_2, CH_4, and N_2 at 1 bar. [b](Al-P)/(Si+Al+P) and Al/(Si+Al) ratios for each SAPO and aluminosilicate materials, respectively.

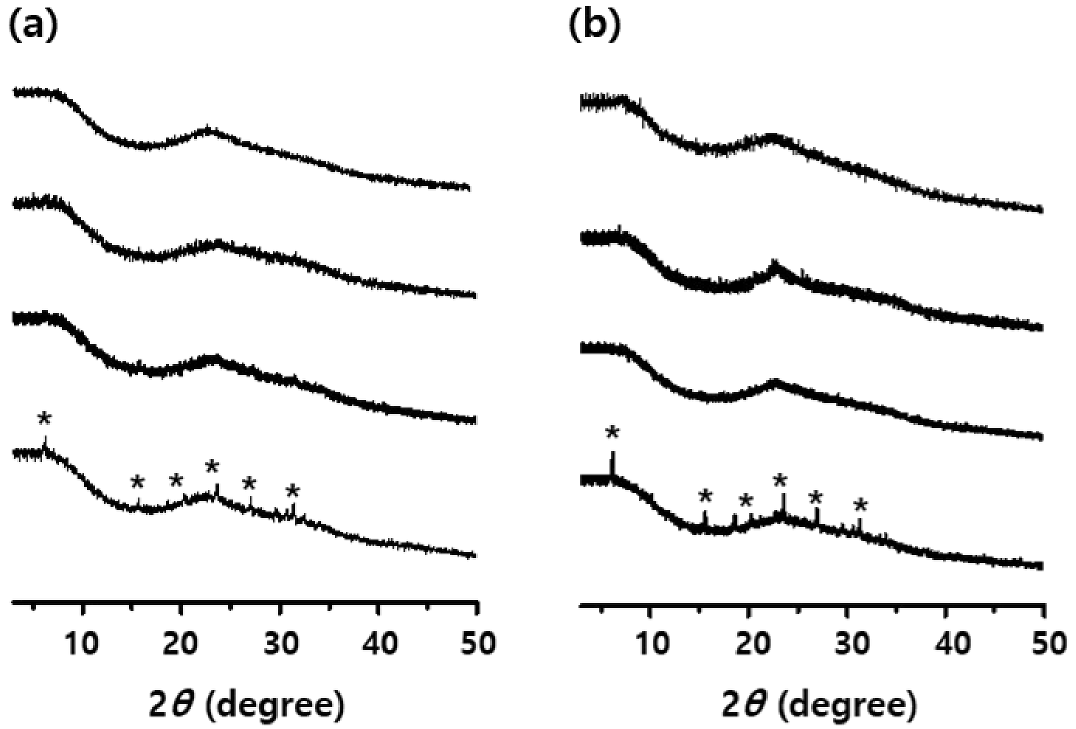

Fig. S1. Powder X-ray diffraction (PXRD) patterns of the solids isolated after aging a synthesis mixture with the chemical composition $4.0NaOH \cdot 1.0Al_2O_3 \cdot 0.9P_2O_5 \cdot 1.0SiO_2 \cdot 150H_2O$ for Na-SAPO-FAU/EMT formation (run no. 14 in Table 1), where a small amount (4 wt% of the alumina in the gel) of (a) NaY (Si/Al = 2.6) or (b) SAPO-37 (Si/(Si+Al+P) = 0.13) was added as seed crystals, respectively, at room temperature for different times (from bottom to top; 0, 12, and 24 h). The top traces of each panel are the PXRD patterns of the solid products obtained after 24 h of heating under static conditions at 110 °C. The representative X-ray peaks due to the FAU structure are marked by asterisks. It can be seen that no X-ray peaks from seed crystals with the FAU structure are detectable by PXRD when aged at room temperature for 12 h. This indicates the digestion of added seed crystals occurs prior to the formation of Na-SAPO-FAU/EMT crystals.

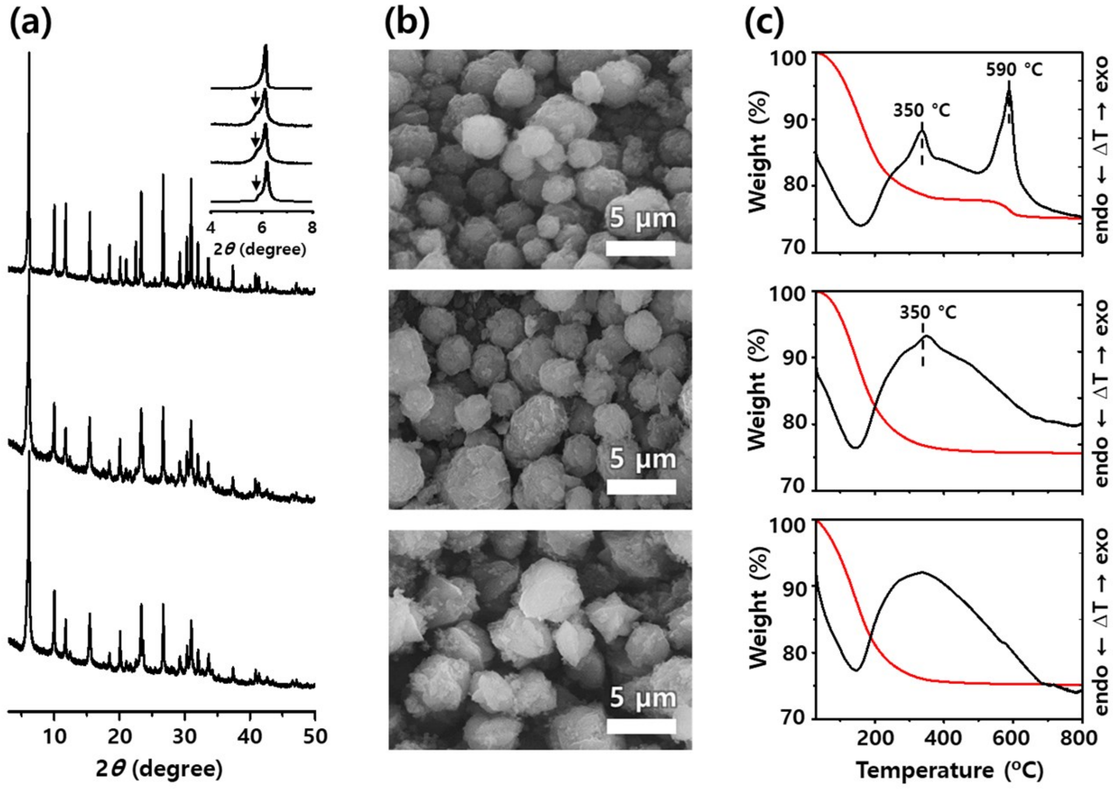

Fig. S2. (a) PXRD patterns, (b) SEM images, and (c) TGA/DTA curves of as-synthesized (from bottom to top) Na-SAPO-FAU/EMT, NaTEA-SAPO-FAU/EMT, and NaTMA-SAPO-FAU. The inset in Fig. S2(a) compares the experimental PXRD patterns (from bottom to top among the top three traces) in the 2θ region 4-8° of Na-SAPO-FAU/EMT, NaTEA-SAPO-FAU/EMT, and NaTMA-SAPO-FAU with the simulated one (bottom) of an FAU/EMT intergrowth structure at a ratio of 80:20[S1,S2]: The arrow indicates the (100) reflection of the EMT structure. We also note that the intergrowth rate of NaTEA-SAPO-FAU/EMT is similar to that of NaTMA-SAPO-FAU/EMT. The TMA cations employed in the synthesis of NaTMA-SAPO-FAU were found to be within both the *fau* and *sod* cages of the FAU structure. The TEA cations, on the other hand, were too large to be present in the *sod* cages of NaTEA-SAPO-FAU/EMT, like the case of SAPO-37.[S3]

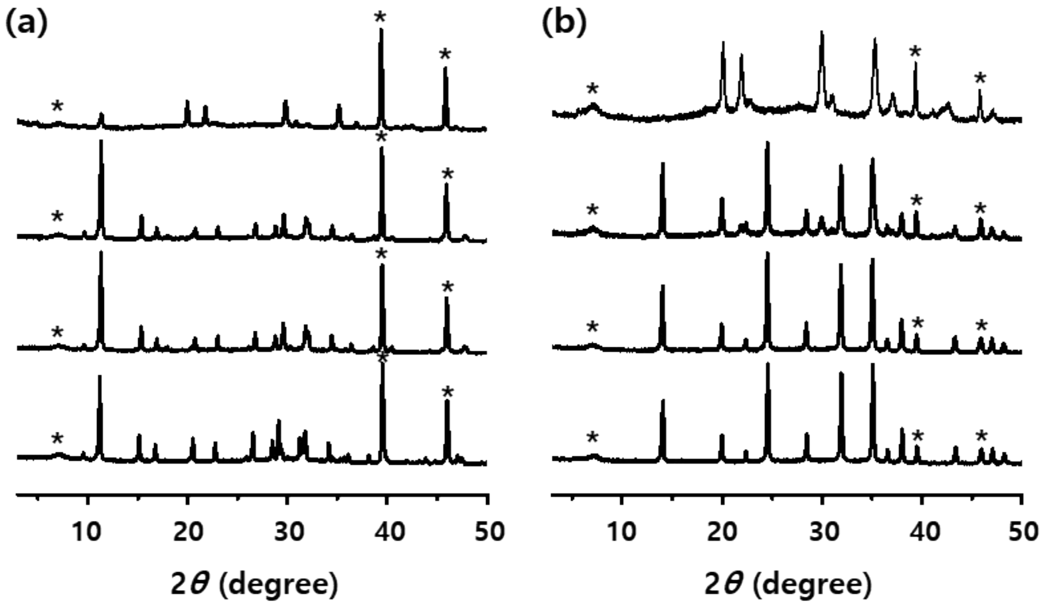

Fig. S3. Variable-temperature (VT) PXRD patterns of as-synthesized (a) Na-ZnAPO-CZP(34) and (b) Na-ZnAPO-SOD(35). Each PXRD pattern was recorded at (from bottom to top) 25, 100, 200, and 300 °C under a residual pressure of 10^{-2} Torr. We note that once the temperature is raised above 300 °C, the structural integrity of both ZnAPO molecular sieves was not maintained. This holds true even after cooling down to 25 °C, unlike the case of SAPO materials prepared in this work (See Fig. S4). The X-ray peaks from the (Pt) sample holder are marked by asterisks.

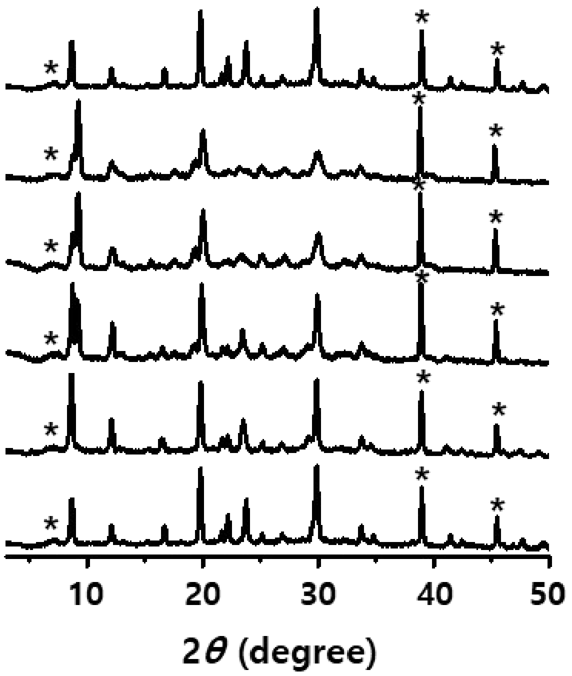

Fig. S4. VT PXRD patterns of as-synthesized Na-SAPO-CHA(17). Each PXRD pattern was obtained at (from bottom to top) 25, 100, 200, 300, and 400 °C under a residual pressure of 10^{-2} Torr. The top pattern, collected at 25 °C under ambient pressure after the thermal process, demonstrates that this thermal treatment is not detrimental to the structural integrity of Na-SAPO-CHA(17). However, notable changes in the position and relative intensity of some X-ray peaks are observed during heating from 25 to 400 °C. This suggests that a phase transition is caused by dehydration at 300 °C, which is under investigation in our laboratory. However, the other SAPO molecular sieves (i.e., Na-SAPO-LTA(11), Na-SAPO-FAU/EMT(14), and NaTMA-SAPO-FAU(15) showed no detectable changes in their PXRD pattern when subjected to the same thermal treatment conditions. The X-ray peaks from the (Pt) sample holder are marked by asterisks.

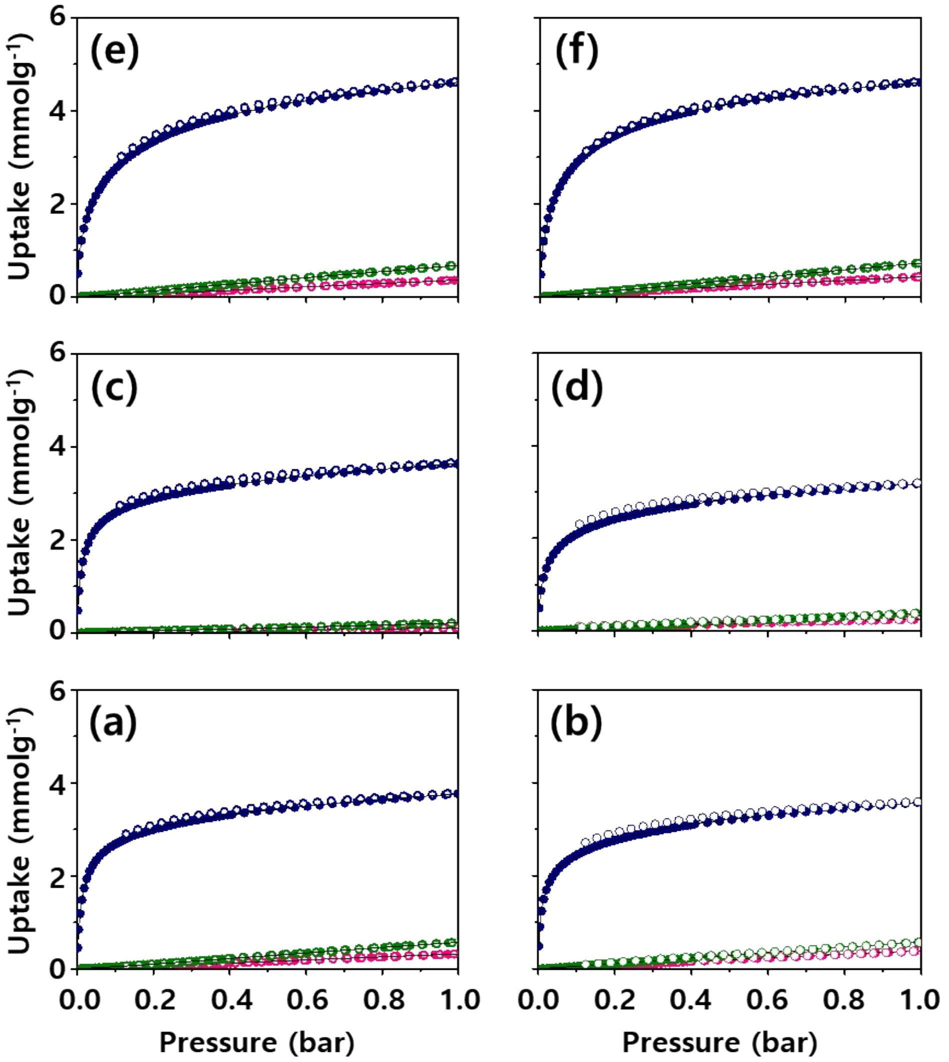

Fig. S5. Small gas adsorption isotherms of the SAPO molecular sieves. CO_2 (blue), N_2 (pink), and CH_4 (green) isotherms at 25 °C were recorded on the Na^+-forms of (a) Na-SAPO-LTA(1), (b) Na-SAPO-LTA(4), (c) Na-SAPO-CHA(17), (d) Na-SAPO-CHA(18), (e) Na-SAPO-FAU/EMT(14), and (f) NaTMA-SAPO-FAU(15).

References

S1 C. Baerlocher and L. B. McCusker, Database of Zeolite Structures, http://www.iza-structure.org/databases/, accessed March 31, 2021.

S2 M. M. J. Treacy, D. E. W. Vaughan, K. G. Strohmaier, J. M. Newsam, *Proc. R. Soc. Lond. A* 1996, **452**, 813.

S3 M. Briend, A. Lamy, M.-J. Peltr, P. P. Man, D. Barthomeuf, *Zeolites* 1993, **13,** 201.
